# Optimization of an Inclusion Body-Based Production of the Influenza Virus Neuraminidase in *Escherichia coli*

**DOI:** 10.3390/biom12020331

**Published:** 2022-02-19

**Authors:** Sabina Lipničanová, Barbora Legerská, Daniela Chmelová, Miroslav Ondrejovič, Stanislav Miertuš

**Affiliations:** 1 Department of Biotechnology, Faculty of Natural Sciences, University of SS. Cyril and Methodius, J. Herdu 2, SK-91701 Trnava, Slovakia; slipnicanova@gmail.com (S.L.); barbora.legerska@ucm.sk (B.L.); daniela.chmelova@ucm.sk (D.C.); icarst.miertus@libero.it (S.M.); 2 International Centre for Applied Research and Sustainable Technology n.o., Jamnického 19, SK-84101 Bratislava, Slovakia

**Keywords:** neuraminidase, inclusion body, recombinant protein, expression, optimization, response surface methodology

## Abstract

Neuraminidase (NA), as an important protein of influenza virus, represents a promising target for the development of new antiviral agents for the treatment and prevention of influenza A and B. Bacterial host strain *Escherichia coli* BL21 (DE3)pLysS containing the NA gene of the H1N1 influenza virus produced this overexpressed enzyme in the insoluble fraction of cells in the form of inclusion bodies. The aim of this work was to investigate the effect of independent variables (propagation time, isopropyl *β*-d-1-thiogalactopyranoside (IPTG) concentration and expression time) on NA accumulation in inclusion bodies and to optimize these conditions by response surface methodology (RSM). The maximum yield of NA (112.97 ± 2.82 U/g) was achieved under optimal conditions, namely, a propagation time of 7.72 h, IPTG concentration of 1.82 mM and gene expression time of 7.35 h. This study demonstrated that bacterially expressed NA was enzymatically active.

## 1. Introduction

Influenza is a respiratory disease caused by a virus belonging to the family *Orthomyxoviridae* [1]. Every year, influenza viruses cause seasonal epidemics that mainly affect the adult population. Of the total number of infected adults, 10–30% are hospitalized, and 3–15% of those infected die [2,3]. Influenza pandemics including the Spanish flu (1918), Asian flu (1957) or Hong Kong flu (1968) have killed millions of people [4,5]. Mortality from the highly symptomatic H5N1 influenza virus in 1997 exceeded 60% [6,7]. Influenza virus variability can lead to a pandemic posing a serious threat to public health [8,9].

Vaccination currently remains the most effective tool for influenza infection prevention [10]. However, influenza viruses tend to mutate, and the vaccine needs to be updated frequently. Therefore, there is an effort to develop antiviral drugs that effectively suppress the infection. Research has mainly been focused on neuraminidase (NA) inhibition [11,12,13,14,15,16,17]. NA is a sialidase [18] and helps to release new virions from infected cells. It is possible to protect the host and prevent the multiplication of viruses in the body by the inhibition of this enzyme [19]. Some NA inhibitors are already available on the market, such as oseltamivir (Tamiflu), zanamivir (Relenza), peramivir (Rapivab) and laninamivir (Inavir) [20,21,22,23]. These classical NA inhibitors are mostly polar and have poor oral bioavailability. In addition, some mutants (e.g., A (H1N1)pdm09) can be resistant to these inhibitors, and have shown strong resistance to oseltamivir [24].

There is a need to have a broad spectrum of different types of NA to effectively study new compounds with the potential to inhibit viral NA. NAs can be extracted directly from the virus surface or recombinantly produced using a suitable host [25]. Although NAs are commonly obtained from the surface of the virus, this process requires expensive laboratory equipment and adherence to laws covered by health and safety legislation [26,27]. It is also necessary to obtain large amounts of enzyme to test inhibitors, so it is more convenient to produce NA recombinantly. Of all the options tested [28,29,30,31], recombinant production of this enzyme in bacteria is the simplest. We found in our previous study [32] that induction of recombinant *Escherichia coli* carrying the viral NA gene resulted in the formation of inclusion bodies independent of the selected conditions. Although the inclusion bodies also contain waste products of bacteria [33], the selection of suitable conditions for the solubilization of the inclusion bodies led to the release of the biologically active NA [34]. The biological activity of NA was confirmed using fetuin as a substrate [32]. Moreover, the optimization of production conditions such as inducer concentration or expression time can lead to higher yields of target proteins in inclusion bodies [35].

The aim of this study was to investigate the influence of factors such as propagation time, isopropyl *β*-d-1-thiogalactopyranoside (IPTG) concentration, expression time and temperature, which affect the NA yield in an insoluble fraction, in the form of inclusion bodies. Subsequently, the optimization was performed using response surface methodology (RSM) to maximize NA production.

## 2. Materials and Methods

### 2.1. Chemicals and Equipment

Chemicals were obtained from Sigma-Aldrich (Taufkirchen, DE), Mikrochem (Pezinok, SK), Biomark Laboratories (Maharashtra, IN), Difco Laboratories (Detroit, MI, USA), Serva Electrophoresis (Heidelberg, DE), Biolife (Milan, IT), ThermoFisher Scientific (Waltham, MA, USA) and AppliChem (Darmstadt, DE). A Sonopuls HD 2200 equipped with VS 70T probe (Bandelin electronic, Berlin, Germany) was used to disrupt cell membranes and release cell contents. The SE250 mini vertical protein electrophoresis unit (Hoefer, Holliston, MA, USA) was used for SDS-PAGE (sodium dodecyl sulfate polyacrylamide gel electrophoresis). All spectrophotometric measurements were performed using an EL × 800 absorbance reader (BioTek Instruments, Winooski, VT, USA). 

### 2.2. Bacterial Strain, Plasmid and Growth Conditions

The NA gene was designed according to the codon usage of *E. coli* using the sequence deposited on GenBank under the accession number KM244086.1 and synthetized in the pET15b vector (ATG Biosynthetic, Merzhausen, DE). *E. coli* expression strain BL21 (DE3)pLysS was purchased from Agilent Technologies (Santa Clara, CA, USA) and transformed by pET15b containing the NA gene according to a standard protocol. The presence of the NA gene was verified by restriction digestion using *XhoI* and *NdeI* restriction enzymes (ThermoFisher Scientific, Waltham, MA, USA) after transformation of the *E. coli* strain [32].

Luria Bertani broth (10 g/L tryptone, 10 g/L NaCl and 5 g/L yeast extract) with ampicillin (100 μg/mL) was inoculated with a single bacterial colony of transformed *E. coli* BL21 (DE3)pLysS and the culture was incubated overnight at 37 °C under shaking (200 RPM). This overnight culture (0.5 mL) was used to prepare a stock containing glycerol at a final concentration of 25% (*v/v*). Glycerin stocks were stored in a freezer (Arctiko ULUF, Esbjerg Kommune, DK) at −80 °C and used to prepare the inoculum (25 mL).

The inoculum was prepared by mixing LB broth (25 mL supplemented with ampicillin 100 μg/mL) and one glycerin stock. The culture was incubated for 12 h at 37 °C and 200 RPM. The cells were harvested at 3000 RPM for 5 min and then diluted with sterile distilled water to obtain 2.0 McFarland units (MFU) solution. Prepared inoculum was used to inoculate the culture medium at a final concentration of 2% (*v/v*) at different propagation times. The expression of recombinant enzyme was inducted by adding isopropyl β-d-1-thiogalactopyranoside (IPTG) at different concentrations for various time periods.

### 2.3. Selection of Independent Variables

The effect of propagation time (2, 4, 6, 8, 10 and 24 h), IPTG concentration (0, 0.1, 0.5, 1, 1.5 and 2 mM), expression temperature (10, 20, 30, 37 and 45 °C) and time (3, 6, 10, 20 and 48 h) on NA yield was evaluated. The laboratory fermenter (Biostat A plus, Sartorius AG, Göttingen, DE) was filled with a sterile culture medium and inoculated. Normally, NA gene expression was induced after 8 h of propagation by the addition of IPTG (1 mM) for 20 h at 37 °C and 200 RPM. 

After expression, the medium was separated from *E. coli* biomass by centrifugation (4000 RPM for 10 min) and the biomass was processed to release soluble and insoluble cell proteins. Soluble and insoluble cell fractions were analyzed by SDS-PAGE. The pellet was then used to determine biomass yield, isolate inclusion bodies and evaluate the yield of proteins released from inclusion bodies as well as NA yield.

### 2.4. Experimental Design and Optimization

RSM was used to investigate the effect of propagation time, IPTG concentration and expression time on the dependent variables (protein yield and NA yield). These three independent factors were tested on five code levels: −1.682; −1; 0; 1 and 1.682 (Table 1). 

The second-order polynomial function with respect to the three selected parameters is given in Equation (1).

(1) $$Y=b_{0}+\overset{k}{\underset{i=1}{\sum }}b_{i}X_{i}+\overset{k}{\underset{i=1}{\sum }}b_{ii}X_{i}^{2}+\overset{k-1}{\underset{\begin{array}{c} i=1\\ i<j \end{array}}{\sum }}\underset{j=2}{\sum }b_{ij}X_{i}X_{j}\;\;\;$$

where *X* are the independent variables (propagation time, IPTG concentration or expression time) causing the *Y* response (protein yield or NA yield) and *b* are regression coefficients. The interaction between two variables and the effect of these factor levels on the protein or NA yield were derived from 3D surface response plots. The third constant was kept at the optimized point. The coefficients of the response surface equation were estimated.

### 2.5. Isolation of Inclusion Bodies and NA Renaturation

The procedure for isolating inclusion bodies from *E. coli* cells and the subsequent release and renaturation of NA was proposed previously [32]. Briefly, the cell pellet was first resuspended in lysis solution (100 mM NaCl, 5 mM ethylenediaminetetraacetic acid (EDTA), 1 mM phenylmethylsulfonyl fluoride (PMSF), 10 mM DL-dithiothreitol (DTT) and 5 g/L lysozyme in 100 mM tris (hydroxymethyl)aminomethane (Tris)-HCl buffer, pH 8.0) followed by sonication in 10 cycles (30 s sonication alternated with 30 s of incubation on ice). The cell pellets were harvested (4000 RPM for 30 min) and washed 5 times with a solution of urea (2 M) in 100 mM Tris-HCl buffer. Inclusion bodies were obtained by centrifugation (4000 RPM for 30 min) and were then dissolved in a solution of urea (8 M) in 100 mM Tris-HCl (pH 8.0). The suspension was dialyzed against a solution with NaCl (150 mM) and EDTA (5 mM) in 50 mM Tris-HCl to refold the recombinant NA. The refolded and soluble proteins were centrifuged (4000 RPM for 20 min) and the supernatants were used for further analyses.

### 2.6. Analytical Methods

The amount of biomass was determined after centrifugation of the culture medium at 4000 RPM for 20 min, the biomass was washed twice with distilled water, then dried to constant weight at 60 °C and expressed in grams per volume of culture medium.

The concentration of released proteins from the inclusion bodies was determined using the Bradford method [36] with bovine serum albumin as a standard and expressed as protein yield released from the inclusion bodies per gram of dry biomass (mg/g).

SDS-PAGE analysis (80 V, 2.5 h) was carried out using a 12% (*w/v*) separation gel in Tris-glycine buffer (pH 8.3) [37]. After electrophoresis, the gels were stained with CBB-R250 solution and then washed in a destaining solution containing 10% (*v/v*) methanol. NA captured in the gel was analyzed by densitometry using the ImageJ (version 1.46r, National Institute of Health, Bethesda, MD, USA). The amount of NA was evaluated from a linear regression of the calibration curve as a function of the peak area (pixel) on the lysozyme concentration and expressed as the amount of NA per biomass (mg/g).

After recovery of recombinant NA from inclusion bodies [32], enzyme activity was determined by coupled reactions using fetuin as a substrate [32,38]. One unit of refolded NA activity (U) was defined as the amount of enzyme that converts 1 µmol of fetuin per minute at a wavelength of 540 nm and expressed in U/g of bacterial biomass.

### 2.7. Statistical Analysis

OriginPro 2016 (version 9.3, OriginLab Corporation, Northampton, MA, USA) was used to process all experimental data obtained. Statgraphics Centurion XV (version 15.1.2, Statpoint Technologies, Warrenton, VA, USA) was used for the statistical analysis of experimental data. All assays were performed in triplicate.

## 3. Results and Discussion

### 3.1. Preliminary Experiments

In higher eukaryotic expression systems, the expression of the native gene directly amplified from the influenza virus occurs without major problems [39]. However, when such genes are expressed in *E. coli* cells, problems arise due to the different use of individual codons, leading to a protein without biological activity. Regardless of the chosen expression conditions of the inserted NA gene, the recombinant protein accumulates in the insoluble fraction of the cell biomass in the form of inclusion bodies. The formation of inclusion bodies is a common but often undesirable phenomenon associated with the overexpression of a heterologous gene in *E. coli* cells. However, accumulation of recombinant protein in inclusion bodies may be an advantage. They contain a high proportion of target protein and at the same time fewer undesirable proteins [40,41,42]. The optimization of recombinant proteins expressed in this form is relatively rare, but may represent a promising strategy for various insoluble proteins produced by *E. coli* [35,43,44,45,46]. In our previous work [26], we were able to produce biologically active NA in *E. coli* cells, and now we bring our focus to optimizing the conditions for the production of recombinant enzymes in inclusion bodies in order to maximize NA production.

**Figure 1:**
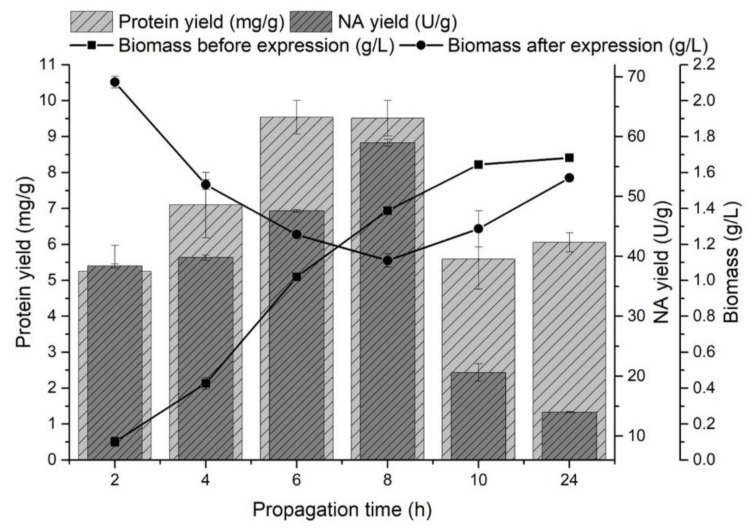
The effect of producer propagation time on the protein yield (mg/g) released from inclusion bodies, the NA yield (U/g) and on the amount of biomass (g/L) before and after NA gene expression.

The effect of propagation (pre-incubation) time on the monitored variables was observed in the range of 2–24 h at 37 °C. Recombinant protein production was induced by the addition of a 1 mM IPTG solution at the end of the lag phase (biomass yield 0.10 ± 0.03 g/L), in the early exponential phase (0.43 ± 0.03 g/L), in the mid-exponential phase (1.02 ± 0.02 g/L), in the late exponential phase (1.39 ± 0.02 g/L), in the early stationary phase (1.64 ± 0.02 g/L) and in the stationary phase (1.68 ± 0.03 g/L) of producer growth (Figure 1). 

A significant increase in biomass yield was observed after the NA gene expression at the end of the lag phase and at the beginning of the exponential growth phase. The highest yields of NA released from the inclusion bodies were obtained by induction in the mid-exponential phase of growth (47.58 ± 0.22 U/g) and at the end of this phase (58.97 ± 0.58 U/g), and protein yields were comparable (9.54 ± 0.47 mg/g and 9.51 ± 0.49 mg/g, respectively). Fazaeli et al. [47] also observed the highest recombinant enzyme production in the mid-exponential phase of growth. The highest NA yields were obtained by inducing *E. coli* cells during the exponential phase of growth (Figure 1). A decrease in biomass growth and a higher yield of proteins released from inclusion bodies could indicate a producer’s transition from biomass production to recombinant protein production [48]. Most preferably, the late exponential phase of growth induced gene expression (31.87 ± 0.31 U/g). Similarly, Rengby et al. [49] found that changing the induction time from mid-exponential to late exponential growth phase increased the recombinant protein yield 4-fold.

**Figure 2:**
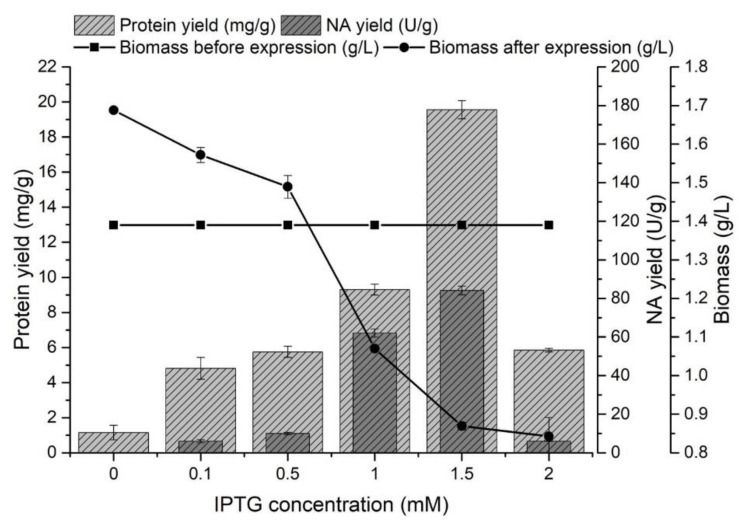
The effect of IPTG concentration on the protein yield (mg/g) released from inclusion bodies, the NA yield (U/g) and on the amount of biomass (g/L) before and after NA gene expression.

The effect of IPTG concentration on the protein and NA yields from inclusion bodies was monitored in the range of 0–2 mM. The producer was cultivated for 8 h at 37 °C, and after reaching the late exponential growth phase (biomass yield 1.39 ± 0.02 g/L), gene expression was started by adding IPTG. In all experiments (Figure 2), the presence of proteins released from inclusion bodies was determined. 

The presence of proteins in the inclusion bodies was also determined in medium without IPTG, but the presence of NA was not confirmed after expression. The increasing concentration of IPTG caused a higher expression of NA, but it had a negative effect on the amount of biomass. This effect has also been described in other studies [50,51]. The highest protein yield released from the inclusion bodies (19.56 ± 0.52 mg/g) was achieved by adding IPTG at a concentration of 1.5 mM. Increasing the IPTG concentration from 1 to 1.5 mM caused a 1.4-fold higher enzyme yield (84.09 ± 3.21 U/g) at a concentration of 1.5 mM IPTG. 

The effect of expression time in the range of 0–48 h and expression temperature of 11–45 °C on the monitored variables was evaluated after 8 h of propagation, and NA expression was induced by 1.5 mM IPTG (data not shown). *E. coli* cells can grow over a wide temperature range (15–45 °C) with optimal growth in the range of 20–42 °C. A direct relationship between the peptide chain elongation rate of the newly formed protein and temperature during expression of the corresponding gene has been observed in this temperature range [51,52]. We found that temperatures of 11 and 45 °C were not suitable for NA expression. At 11 °C, the lowest protein and NA yields were obtained. The temperature of 45 °C caused partial denaturation of the undesirable proteins but also the recombinant NA. Protein yields were highest at 20–37 °C after a 20 h expression time (21.24–23.61 mg/g) and did not change significantly after this time. However, the NA yield at 37 °C was 1.3 times higher than at 30 °C, and 2.3 times higher than at 20 °C. The NA yield did not change significantly after 10 h of expression (84.57 ± 5.12 U/g). Therefore, we set the expression temperature to 37 °C during the optimization and the extraction time was chosen as an independent variable.

### 3.2. Optimization of Neuraminidase Production in Inclusion Bodies Using Response Surface Methodology

Preliminary experiments confirmed that parameters such as propagation time, IPTG concentration and gene expression time at 37 °C affected the protein yield from inclusion bodies and the NA yield itself. The optimal values of these selected production parameters were calculated using RSM in order to obtain the maximum yield of NA produced by *E. coli*. Table 2 shows the design matrix including coded and actual variables as well as the protein and NA yields for each run (1–17). 

**Figure 3:**
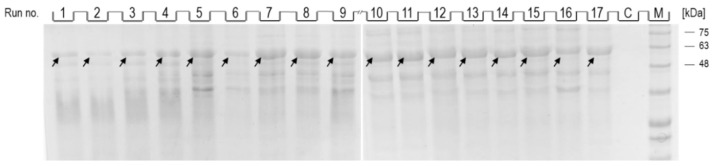
SDS-PAGE analysis of dissolved inclusion body fractions from individual optimization runs (1–17) and control (C). The position of the band corresponding to the recombinant NA in each lane is indicated by an arrow. Protein ladder indicates molecular weights in kDa (lane M).

As shown in Figure 3, after dissolution of inclusion bodies obtained from induced cells from all optimization runs (1–17), a band corresponding to NA (indicated by an arrow; 54 kDa) could be observed, while no band corresponding to NA was observed in the control (Figure 3, lane C). The intensity of the individual bands varied depending on the conditions of NA production, and while the most intense bands could be observed in runs 7–11, the intensity of the bands from runs 1, 2 and 6 was the lowest.

**Figure 4:**
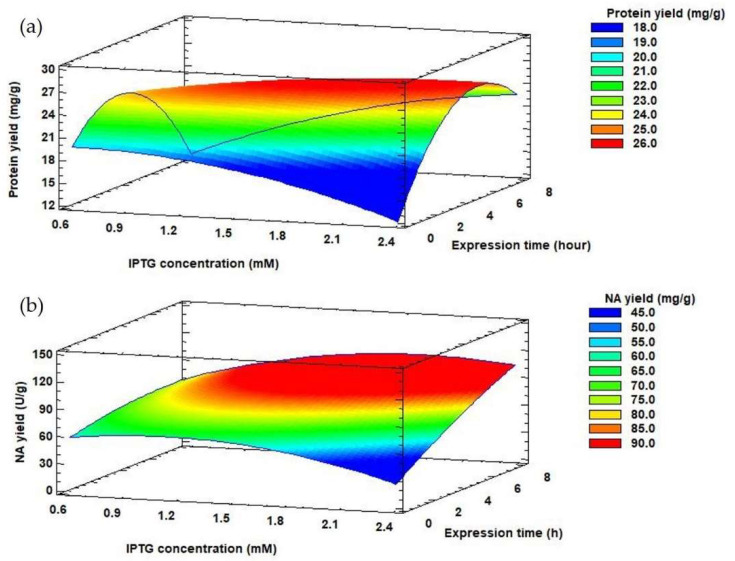
Response surface model plots showing interaction effects between IPTG concentration and expression time on protein yield (**a**) and NA yield (**b**) at a constant optimal value of propagation time.

Depending on the selected production conditions, the protein yield released from the inclusion bodies also varied, in the range of 18.52–28.84 mg/g. The highest protein yield released from the inclusion bodies (28.84 ± 1.01 mg/g) was achieved by 4.7 h propagation of the producer and gene expression of 1.5 mM IPTG for 4 h. However, in terms of NA yield, it is more appropriate to extend the propagation and expression time to 8 and 7.3 h, respectively with 1.5 mM IPTG concentration (Table 1) (124.06 ± 2.91 U/g). *E. coli* cells were held in the late exponential phase of growth by prolonging the propagation time. This growth phase appears to be most suitable for the synthesis of other recombinant proteins, such as human betaferon and heat shock protein (HSPA6) [51,53]. The effect of IPTG concentration and expression time on protein and NA yields can be monitored using response surface model plots (Figure 4) at a constant value of optimized propagation time (7.72 h).

**Figure 5:**
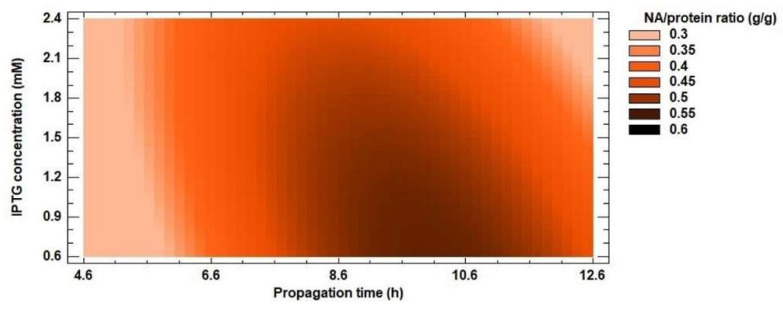
Response surface model plot showing the effect of IPTG concentration and propagation time on NA/protein ratio (g/g) at a constant optimized value of propagation time.

The experimentally measured data were evaluated by a second-order polynomial model (Equation 1) for protein and NA yields. Inclusion bodies usually contain almost exclusively overexpressed recombinant protein [54,55,56], but the results of the optimization do not confirm this. For protein yield, the *R^2^* coefficient reached 79% and the calculated *R^2^* value of the model for NA yield was 94%. These results suggest that recombinant protein was not the only protein found in inclusion bodies. The ratio of NA amount (g/g) in inclusion bodies and protein yield (g/g) ranged from 16 to 60% (Figure 5). The lowest value was reached at the shortest propagation time, the lowest IPTG concentration and the shortest expression time from the optimization matrix. As the values of the independent variables increased, so did the amount of recombinant enzyme in the insoluble fraction. Dang et al. [57] found that inclusion bodies contained approximately 80% recombinant protein. Therefore, we continued to work only on optimizing the conditions for NA yield.

Table 3 summarizes the regression coefficients and analysis of variances calculated for NA yield. For NA yield, the propagation time and the expression time had a significant positive linear influence (*p*-value < 0.05). The increase of the expression time from 4 to 7 h affected the value of NA yield (Figure 4A). Moreover, the propagation time had a negative quadratic influence (*p*-value < 0.05) on NA yield (Table 3). 

The optimal production conditions for the highest NA yield were propagation time 7.72 h, IPTG concentration 1.82 mM and expression time 7.25 h. Optimal production conditions were verified and there was no significant difference between predicted and experimental values of NA yield (*p* < 0.05). Our results indicate that properly set optimization can increase the yield of the recombinant protein. The NA yield was increased 1.9-fold from the original 58.97 ± 0.58 (Figure 1) to 112.97 ± 2.82 U/g (Table 4).

Moreover, here we confirmed the results of our previous paper [32] where the refolded non-glycosylated monomer of NA produced by *E. coli* at optimal production conditions achieved V_max_ value of 9.73 U/mg with k_cat_ 8.76 s^−1^ and the affinity to fetuin was demonstrated by the K_m_ value of 0.51 g/L.

## 4. Conclusions

In this study, we focused on the optimization of the expression of influenza virus NA, the enzyme responsible for releasing new virions from infected cells. We tested various expression conditions, such as propagation time, IPTG concentration and expression temperature and time. The recombinant enzyme production process was optimized using response surface methodology, which led to a higher production of NA in inclusion bodies. The optimal values were as follows: propagation time 7.72 h, IPTG concentration 1.82 mM and gene expression time 7.35 h. The maximum NA yield was 112.97 ± 2.82 U/g, resulting in a 1.9-fold increase over the original production conditions. The results of this study confirmed that influenza virus NA can be produced by *E. coli* cells. 

## Figures and Tables

**Table 1:** Interpretation of coded levels of the three independent variables tested by RSM.

Variables	Code Levels				
	−1.682	−1	0	1	1.682
Propagation time (h)	4.653	6	8	10	11.347
IPTG concentration (mM)	0.663	1	1.5	2	2.337
Expression time (h)	0.653	2	4	6	7.347

**Table 2:** Experimental design with actual and coded levels of independent variables as well as the measured protein (mg/g) and NA (U/g) yields.

Run no.	Propagation Time	IPTG Concentration	Expression Time	Protein Yield	NA Yield
	(h)	(mM)	(h)	(mg/g)	(U/g)
1	10.0 (1)	1.0 (−1)	6.0 (1)	19.52 ± 1.44	74.43 ± 1.82
2	6.0 (−1)	1.0 (−1)	2.0 (−1)	22.49 ± 1.61	26.37 ± 1.01
3	8.0 (0)	1.5 (0)	4.0 (0)	27.99 ± 2.38	83.29 ± 2.44
4	10.0 (1)	2.0 (1)	2.0 (−1)	21.73 ± 0.85	57.02 ± 1.21
5	6.0 (−1)	2.0 (1)	6.0 (1)	26.66 ± 1.21	67.14 ± 2.02
6	6.0 (−1)	2.0 (1)	2.0 (−1)	18.52 ± 0.24	37.46 ± 1.23
7	10.0 (1)	1.0 (−1)	2.0 (−1)	26.17 ± 0.12	88.54 ± 1.40
8	10.0 (1)	2.0 (1)	6.0 (1)	21.49 ± 0.79	80.47 ± 0.72
9	6.0 (−1)	1.0 (−1)	6.0 (1)	27.24 ± 2.71	57.31 ± 2.12
10	8.0 (0)	1.5 (0)	4.0 (0)	24.85 ± 1.99	92.92 ± 3.71
11	8.0 (0)	1.5 (0)	7.347 (1.682)	21.09 ± 3.71	124.06 ± 2.91
12	8.0 (0)	0.663 (−1.682)	4.0 (0)	21.35 ± 1.34	89.03 ± 4.03
13	8.0 (0)	2.337 (1.682)	4.0 (0)	28.23 ± 1.27	85.43 ± 2.15
14	8.0 (0)	1.5 (0)	0.653 (−1.682)	21.03 ± 1.42	76.09 ± 1.91
15	8.0 (0)	1.5 (0)	4.0 (0)	25.54 ± 1.14	95.65 ± 2.44
16	4.653 (−1.682)	1.5 (0)	4.0 (0)	28.84 ± 1.01	52.25 ± 1.23
17	11.347 (1.682)	1.5 (0)	4.0 (0)	20.70 ± 1.49	66.85 ± 3.20

Independent variables coded in different levels (−1 and +1), with a central point (0) and two axial points (−1.682 and 1.682).

**Table 3:** Regression coefficients of the predicted second-order polynomial models for NA yield.

Effect	Factor ^1^	NA yield
Constant		−373.5050
Linear	A	**83.2431**
	B	97.3955
	C	**14.1455**
Quadratic	AA	−**3.9444**
	BB	−23.5673
	CC	−0.3261
Interaction	AB	−5.8000
	AC	−1.6025
	BC	4.5375

^1^ A—propagation time, B—IPTG concentration and C—expression time. Statistically significant differences at *p*-value < 0.05 are shown in bold.

**Table 4:** Predicted and experimentally verified values of NA yield under optimal production conditions.

Value	NA Yield (U/g)
Predicted	107.85
Experimental	112.97 ± 2.82
Precision (%)	95.47

## References

[1] Taubenberger J.K., Morens D.M. (2006). 1918 Influenza: The mother of all pandemics. Emerg. Infect. Dis..

[2] Li G., Yılmaz M., Kojicic M.V., Fernández-Pérez E., Wahab R., Huskins W.C., Afessa B., Truwit J.D., Gajic O., Yilmaz M. (2009). Outcome of critically ill patients with influenza virus infection. J. Clin. Virol..

[3] Mauskopf J., Klesse M., Lee S., Herrera-Taracena G. (2012). The burden of influenza complications in different high-risk groups: A targeted literature review. J. Med. Econ..

[4] Patterson K.D., Pyle G.F. (1991). The geography and mortality of the 1918 influenza pandemic. Bull. Hist. Med..

[5] Cox N.J., Subbarao K. (2000). Global Epidemiology of Influenza: Past and Present. Annu. Rev. Med..

[6] Claas E.C., Osterhaus A.D., van Beek R., De Jong J.C., Rimmelzwaan G.F., Senne D.A., Krauss S., Shortridge K.F., Webster R.G. (1998). Human influenza A H5N1 virus related to a highly pathogenic avian influenza virus. Lancet.

[7] Khurana S., Sasono P., Fox A., Van Kinh N., Mai L.Q., Thai P.Q., Hien N.T., Liem N.T., Horby P., Golding H. (2011). H5N1-SeroDetect EIA and Rapid Test: A Novel Differential Diagnostic Assay for Serodiagnosis of H5N1 Infections and Surveillance. J. Virol..

[8] Yoshida R., Igarashi M., Ozaki H., Kishida N., Tomabechi D., Kida H., Ito K., Takada A. (2009). Cross-Protective Potential of a Novel Monoclonal Antibody Directed against Antigenic Site B of the Hemagglutinin of Influenza A Viruses. PLOS Pathog..

[9] Krammer F., Smith G.J.D., Fouchier R.A.M., Peiris M., Kedzierska K., Doherty P.C., Palese P., Shaw M.L., Treanor J., Webster R.G. (2018). Influenza. Nat. Rev. Dis. Primers.

[10] Bassetti M., Castaldo N., Carnelutti A. (2019). Neuraminidase inhibitors as a strategy for influenza treatment: Pros, cons and future perspectives. Expert Opin. Pharmacother..

[11] Kongkamner J., Cappelletti L., Prandi A., Seneci P., Rungrotmongkol T., Jongaroonngamsang N., Rojsitthisak P., Frecer V., Milani A., Cattoli G. (2012). Synthesis and in vitro study of novel neuraminidase inhibitors against avian influenza virus. Bioorg. Med. Chem..

[12] Udommaneethanakit T., Rungrotmongkol T., Frecer V., Seneci P., Miertus S., Bren U. (2014). Drugs Against Avian Influenza A Virus: Design of Novel Sulfonate Inhibitors of Neuraminidase N1. Curr. Pharm. Des..

[13] Hľasová Z., Košík I., Ondrejovič M., Miertuš S., Katrlík J. (2019). Methods and current trends in determination of neuraminidase activity and evaluation of neuraminidase inhibitors. Crit. Rev. Anal. Chem..

[14] Shie J.-J., Fang J.-M. (2019). Development of effective anti-influenza drugs: Congeners and conjugates—A review. J. Biomed. Sci..

[15] Woo H.S., Shin K.-C., Kim J.Y., Kim Y.-S., Ban Y.J., Oh Y.J., Cho H.J., Oh D.-K., Kim D.W. (2020). Bakkenolides and Caffeoylquinic Acids from the Aerial Portion of *Petasites japonicus* and Their Bacterial Neuraminidase Inhibition Ability. Biomolecules.

[16] Ha T.K.Q., Lee B.W., Nguyen N.H., Cho H.M., Venkatesan T., Doan T.P., Kim E., Oh W.K. (2020). Antiviral Activities of Compounds Isolated from *Pinus densiflora* (Pine Tree) against the Influenza A Virus. Biomolecules.

[17] Mahal A., Duan M., Zinad D.S., Mohapatra R.K., Obaidullah A.J., Wei X., Pradhan M.K., Das D., Kandi V., Zinad H.S. (2021). Recent progress in chemical approaches for the development of novel neuraminidase inhibitors. RSC Adv..

[18] Lipničanová S., Chmelová D., Ondrejovič M., Frecer V., Miertuš S. (2020). Diversity of sialidases found in the human body—A review. Int. J. Biol. Macromol..

[19] Mitrasinovic P.M. (2010). Advances in the structure-based design of the influenza A neuraminidase inhibitors. Curr. Drug Targets.

[20] Dunn C.J., Goa K.L. (1999). Zanamivir: A review of its use in Influenza. Drugs.

[21] McClellan K., Perry C.M. (2001). Oseltamivir: A review of its use in influenza. Drugs.

[22] Kubo S., Tomozawa T., Kakuta M., Tokumitsu A., Yamashita M. (2010). Laninamivir prodrug CS-8958, a long-acting neuraminidase inhibitor, shows superior antiinfluenza virus activity after a single administration. Antimicrob. Agents Chemother..

[23] Anuwongcharoen N., Shoombuatong W., Tantimongcolwat T., Prachayasittikul V., Nantasenamat C. (2016). Exploring the chemical space of influenza neuraminidase inhibitors. PeerJ.

[24] Gubareva L.V., Fallows E., Mishin V.P., Hodges E., Brooks A., Barnes J., Fry A.M., Kramp W., Shively R.E., Wentworth D. (2017). Monitoring influenza virus susceptibility to oseltamivir using a new rapid assay, iART. Eurosurveillance.

[25] Nivitchanyong T., Yongkiettrakul S., Kramyu J., Pannengpetch S., Wanasen N. (2011). Enhanced expression of secretable influenza virus neuraminidase in suspension mammalian cells by influenza virus nonstructural protein 1. J. Virol. Methods.

[26] Wanitchang A., Narkpuk J., Jaruampornpan P., Jengarn J., Jongkaewwattana A. (2012). Inhibition of influenza A virus replication by influenza B virus nucleoprotein: An insight into interference between influenza A and B viruses. Virology.

[27] Benton D.J., Wharton S.A., Martin S.R., McCauley J.W. (2017). Role of neuraminidase in influenza A(H7N9) virus receptor binding. J. Virol..

[28] Yongkiettrakul S., Boonyapakron K., Jongkaewwattana A., Wanitchang A., Leartsakulpanich U., Chitnumsub P., Eurwilaichitr L., Yuthavong Y. (2009). Avian influenza A/H5N1 neuraminidase expressed in yeast with a functional head domain. J. Virol. Methods.

[29] Pua T.L., Loh H.S., Massawe F., Tan C.S., Omar A.R. (2012). Expression of insoluble influenza neuraminidase type 1 (NA1) protein in tobacco. J. Trop. Life Sci..

[30] Dalakouras T., Smith B.J., Platis D., Cox M.M., Labrou N.E. (2006). Development of recombinant protein-based influenza vaccine: Expression and affinity purification of H1N1 influenza virus neuraminidase. J. Chromatogr. A.

[31] Prevato M., Ferlenghi I., Bonci A., Uematsu Y., Anselmi G., Giusti F., Bertholet S., Legay F., Telford J.L., Settembre E.C. (2015). Expression and characterization of recombinant, tetrameric and enzymatically active influenza neuraminidase for the setup of an enzyme-linked lectin-based assay. PLoS ONE.

[32] Lipničanová S., Chmelová D., Godány A., Ondrejovič M., Miertuš S. (2020). Purification of viral neuraminidase from inclusion bodies produced by recombinant *Escherichia coli*. J. Biotechnol..

[33] Garcia-Fruitos E., Vázquez E., Diez-Gil C., Corchero J.L., Seras J., Ratera I., Veciana J., Villaverde A. (2012). Bacterial inclusion bodies: Making gold from waste. Trends Biotechnol..

[34] Yamaguchi H., Miyazaki M. (2014). Refolding techniques for recovering biologically active recombinant proteins from inclusion bodies. Biomolecules.

[35] Gutiérrez-González M., Farías C., Tello S., Pérez-Etcheverry D., Romero A., Zúñiga R., Ribeiro C.H., Lorenzo-Ferreiro C., Molina M.C. (2019). Optimization of culture conditions for the expression of three different insoluble proteins in *Escherichia coli*. Sci. Rep..

[36] Bradford M.M. (1976). A rapid and sensitive method for the quantitation of microgram quantities of protein utilizing the principle of protein-dye binding. Anal. Biochem..

[37] Laemmli U.K. (1970). Cleavage of Structural Proteins during the Assembly of the Head of Bacteriophage T4. Nature.

[38] Alon R., Bayer E.A., Wilchek M. (1991). A coupled enzyme assay for measurement of sialidase activity. J. Biochem. Biophys. Methods.

[39] Schmidt P., Attwood R.M., Mohr P., Barrett S., McKimm-Breschkin J. (2011). A Generic System for the Expression and Purification of Soluble and Stable Influenza Neuraminidase. PLoS ONE.

[40] Singh A., Upadhyay V., Upadhyay A.K., Singh S.M., Panda A.K. (2015). Protein recovery from inclusion bodies of *Escherichia coli* using mild solubilization process. Microb. Cell Factories.

[41] Lopes A.R., Nihei O.K. (2021). Depression, anxiety and stress symptoms in Brazilian university students during the COVID-19 pandemic: Predictors and association with life satisfaction, psychological well-being and coping strategies. PLoS ONE.

[42] Lipničanová S., Chmelová D., Godány A., Ondrejovič M. (2019). Optimization of medium composition for propagation of recombinant *Escherichia coli*. Nova Biotechnol. Chim..

[43] Patra A.K., Mukhopadhyay R., Mukhija R., Krishnan A., Garg L.C., Panda A.K. (2000). Optimization of Inclusion Body Solubilization and Renaturation of Recombinant Human Growth Hormone from *Escherichia coli*. Protein Expr. Purif..

[44] Kopp J., Slouka C., Strohmer D., Kager J., Spadiut O., Herwig C. (2018). Inclusion Body Bead Size in *E. coli* Controlled by Physiological Feeding. Microorganisms.

[45] Krachmarova E., Ivanov I., Nacheva G. (2020). Nucleic acids in inclusion bodies obtained from *E. coli* cells expressing human interferon-gamma. Microb. Cell Factories.

[46] Sani M., Roslan H.A. (2020). Expression of recombinant alcohol dehydrogenase in *Escherichia coli* strain BL21 (DE3) and in plant *Agrobacterium* transformation of tomato seeds. Curr. J. Appl. Sci. Technol..

[47] Fazaeli A., Golestani A., Lakzaei M., Varaei S.S.R., Aminian M. (2019). Expression optimization, purification, and functional characterization of cholesterol oxidase from *Chromobacterium* sp. DS1. PLoS ONE.

[48] Jevševar S., Gaberc-Porekar V., Fonda I., Podobnik B., Grdadolnik J., Menart V. (2005). Production of Nonclassical Inclusion Bodies from Which Correctly Folded Protein Can Be Extracted. Biotechnol. Prog..

[49] Rengby O., Johansson L., Carlson L.A., Serini E., Vlamis-Gardikas A., Kårsnas P., Arnér E.S.J. (2004). Assessment of Production Conditions for Efficient Use of *Escherichia coli* in High-Yield Heterologous Recombinant Selenoprotein Synthesis. Appl. Environ. Microbiol..

[50] Larentis A.L., Nicolau J.F.M.Q., Esteves G.D.S., Vareschini D.T., De Almeida F.V.R., Dos Reis M.G., Galler R., Medeiros M.A. (2014). Evaluation of pre-induction temperature, cell growth at induction and IPTG concentration on the expression of a leptospiral protein in *E. coli* using shaking flasks and microbioreactor. BMC Res. Notes.

[51] Malik A., Alsenaidy A.M., Elrobh M., Khan W., Alanazi M.S., Bazzi M.D. (2015). Optimization of expression and purification of HSPA6 protein from *Camelus dromedarius* in *E. coli*. Saudi J. Biol. Sci..

[52] Farewell A., Neidhardt F.C. (1998). Effect of temperature on in vivo protein synthetic capacity in *Escherichia coli*. J. Bacteriol..

[53] Maldonado L.M.P., Hernández V.E.B., Medina-Rivero E., de la Rosa A.P.B., Flores J.L.F., Acevedo L.G.O., Rodríguez A.D.L. (2007). Optimization of culture conditions for a synthetic gene expression in *Escherichia coli* using response surface methodology: The case of human interferon beta. Biomol. Eng..

[54] Tsumoto K., Ejima D., Kumagai I., Arakawa T. (2003). Practical considerations in refolding proteins from inclusion bodies. Protein Expr. Purif..

[55] Markossian K.A., Kurganov B.I. (2004). Protein folding, misfolding, and aggregation. Formation of inclusion bodies and ag-gresomes. Biochemistry (Moscow).

[56] Cabrita L.D., Bottomley S.P. (2004). Protein expression and refolding – A practical guide to getting the most out of inclusion bodies. Biotechnol. Annu. Rev..

[57] Dang S., Hong T., Bu D., Tang J., Fan J., Zhang W. (2012). Optimized refolding and characterization of active C-terminal ADAMTS-18 fragment from inclusion bodies of *Escherichia coli*. Protein Expr. Purif..

